# Transmembrane Adaptor Proteins Positively Regulating the Activation of Lymphocytes

**DOI:** 10.4110/in.2009.9.2.53

**Published:** 2009-04-30

**Authors:** Inyoung Park, Yungdae Yun

**Affiliations:** Department of Life Science, Ewha Womans' University, Seoul 120-750, Korea.

**Keywords:** TRAP (transmembrane adaptor protein), LAT (Linker for activation of T cells), LIME (Lck-interacting transmembrane adaptor protein), lymphocyte

## Abstract

Engagement of the immunoreceptors initiates signaling cascades resulting in lymphocyte activation and differentiation to effector cells, which are essential for the elimination of pathogens from the body. For the transduction of these immunoreceptor-mediated signals, several linker proteins termed transmembrane adaptor proteins (TRAPs) were shown to be required. TRAPs serve as platforms for the assembly and membrane targeting of the specific signaling proteins. Among seven TRAPs identified so far, LAT and LIME were shown to act as a positive regulator in TCR-mediated signaling pathways. In this review, we will discuss the functions of LAT and LIME in modulating T cell development, activation and differentiation.

## INTRODUCTION

Lymphocytes respond to various extracellular stimuli mediated by surface molecules such as immunoreceptors (e.g., the T cell receptors; TCR, the B cell receptors; BCR, or the Fc receptors; FcR) and additional coreceptors (e.g., CD28 for T cells and CD19 for B cells). As the earliest immunoreceptor-mediated biochemical events, Src-family protein tyrosine kinases (PTKs) become activated. In turn, activated Src PTKs phosphorylate immunoreceptor tyrosine-based activation motifs (ITAMs) within the cytoplasmic tails of immunoreceptor-associated signaling molecules (e.g., CD3 complexes in T cells and Igα and Igβ in B cells). Subsequently, Syk-family PTKs are recruited through their SH2 domains and activated by phosphorylation by Src-family PTKs (1). Once activated, Syk family PTKs in combination with Src-family PTKs phosphorylate a variety of downstream effector molecules, including transmembrane adaptor proteins (TRAPs). TRAPs contain up to 10 tyrosine-based signaling motifs (TBSM) in the cytoplasmic region. Upon antigen receptor engagement, these tyrosine sites are phosphorylated by Srcand/or Syk-family PTKs and serve as the binding motifs for signaling molecules possessing either SH2 or phosphotyrosine-binding (PTB) domains. In this manner, TRAPs serve as platforms to enhance signaling efficiency by assembling and concentrating signaling components to the plasma membrane proximal sites.

The transmembrane adaptor protein family includes seven members named LAT (The linker for activation of T cells) (2), LIME (Lck-interacting transmembrane adaptor protein) (3,4), LAX (linker for activation if X cells) (5), NTAL/LAB (non T-cell activation liner/linker fir activation of B cells) (6), PAG/Cbp (phosphoprotein associated with glycosphingolipid-enriched domain/Csk-binding protein) (7), SIT (SHP2-interacting TRAP) (8), and TRIM (TCR-interacting molecule) (9). TRAPs have common structural features by possessing a short extracellular domain, a single transmembrane domain, and a long cytoplasmic region with several potential tyrosine phosphorylation sites. In the juxtamembrane portion of their cytoplasmic region, LAT, LIME, NTAL/LAB, and PAG/Cbp possess dicystein motif CXXC (C; Cystein, X; any amino acid) that serves as palmitoylation sites. Palmitoylation of this motif allows the targeting of the transmembrane adaptor proteins to lipid rafts, a specialized region of plasma membrane enriched with other signaling molecules, such as Src-family PTKs (10). On the other hand, LAX, SIT and TRIM, which lack palmitoylation sites, are mainly localized in non-raft region and seem to be involved in signaling cascades in different cellular compartments. In spite of their similar structural features, the functions of TRAPs are relatively specialized depending on their expression patterns and binding partners (Table I).

Through the previous studies using cell lines and primary immune cells isolated from genetically engineered mice, TRAPs were shown to integrate immunoreceptor-mediated signals in either positive or negative manner. Particularly in this review, we will focus on LAT and LIME, which exert positive regulatory functions in T lymphocytes. For the readers of this review, the characteristics of each TRAPs are summarized in Table I.

## LINKER FOR ACTIVATION OF T CELLS (LAT)

**Figure 1:**
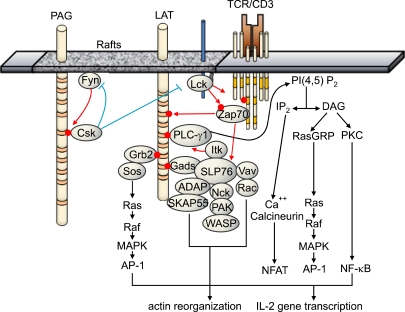
Signaling pathways mediated by LAT. Upon TCR engagement, Src-family kinase, Lck, is activated and phosphorylates the tyrosine residues within ITAM motifs on CD3γ/ε/ζ chains. Subsequently, Syk-family kinase, ZAP-70, is recruited through its SH2 domain and become activated by phosphorylation by Lck. Activated ZAP-70 phosphorylates the tyrosine residues within TBSMs of raft-associated LAT, allowing the recruitment of other signaling molecules possessing SH2 domains or phosphotyrosine-binding (PTB) domains including Gads, Grb2, PLCγ1 at the membrane proximal sites. Through the constitutive interaction with Gads, SLP-76 are recruited to LAT and become phosphorylated by ZAP-70. The recruitment of Itk via SLP-76 is required for full activation of PLCγ1. Activated PLCγ1 hydrolyze the phosphatidylinositol 4, 5 bisphosphate(PIP_2_) to inositol 3, 4, 5-triohosphate (IP_3_) and diacylglycerol (DAG), leading to the calcium mobilization and NF-κB activation. Tyrosine-phosphorylated SLP-76 also provides docking sites for Vav, Nck and ADAP, forming signaling complexes for actin reorganization. Together with Grb2/Sos-mediated Ras/MAPK activation, these signaling pathways cooperatively activate the IL-2 poduction and T cell proliferation. These positive signaling pathways can be negatively regulated by the inhibition of Lck activity by PAG-associated Csk kinase.

LAT was initially identified as a phosphoprotein which is rapidly phosphorylated following TCR ligation (2). The expression of LAT is limited to thymic and peripheral T cells, NK cells, mast cells, megakaryocytes, platelet, and bone-marrow-derived pre-B cells, but not mature B cells and monocytes (2). LAT possesses nine TBSMs in cytoplasmic tail, and becomes phosphorylated at least five tyrosine residues by ZAP-70 or Syk kinases upon immunoreceptor engagement. In addition, LAT is phosphorylated by Itk upon CD28 engagement (11). After phosphorylation, LAT recruits Grb2, Gads, Grap, PLCγ1, p85 PI3K, Vav, 3BP2, and Shb directly via their SH2-domain, and mediates activation of Ras/MAPK, intracellular calcium influx, and cytoskeleton reorganization (12,13). Four distal tyrosines primarily responsible for LAT function are Y132 for PLCγ1, Y171 for PI3K, Y171 and Y191 for Gads, Y191 and Y226 for Vav, Y171, Y191, and Y226 for Grb2, respectively (13). Through the constitutive interaction with Gads, SLP-76 is recruited to LAT upon TCR engagement and in turn, serves as a platform for several signaling molecules such as Vav, NCK, ITK and ADAP. The LAT-SLP-76-PLCγ1 complex formation is required for phosphorylation of PLCγ1, allowing optimal calcium mobilization following TCR ligation. On the other hand, the recruitment of Vav, NCK and ADAP through SLP-76 seems to propagate actin polymerization and integrin activation. In addition, the recruitment of Grb2 is essential in the activation of Ras/MAPK signaling pathway (Fig. 1).

In LAT-deficient Jurkat T cell line, optimal tyrosine phosphorylation of PLCγ1, Vav, and SLP-76 are impaired, resulting in the diminished TCR-mediated calcium mobilization and MAPK activation (14,15). When SLP-76 is targeted constitutively to plasma membrane in LAT-deficient Jurkat T cells, the signaling defects are restored, suggesting that major signal transduction downstream of LAT is mediated by SLP-76 recruited to lipid rafts (16).

Through the extensive studies using genetically engineered mice, it was revealed that LAT is critical in thymocyte development as well as mature T cell activation. In LAT-deficient mice, B cells NK cells, and platelets appear to develop normally, but crucial defects are found in thymocyte development at double-negative stage, resulting in lack of mature peripheral T cell population (17). This suggests that LAT is essential for the pre-TCR signal transduction. Moreover, functional restoration studies using the LAT knock-in mice harboring individual mutations of these tyrosine residues support the importance of LAT in T cell functions. Similar to LAT-deficient mice, mutant mice with point mutations in all distal four tyrosine residues have defects in thymocyte development (18). LAT Y132F mice which possess a mutation in PLCγ1 binding site also have partial defects in thymocyte development in DN stage (19,20). Unexpectedly, however, Y132F mice develop autoimmune phenotype with augmented Th2-type cytokines and B cell proliferation. These results suggest that LAT may also function as a negative modulator in lymphocytes.

As previously indicated, LAT is palmitoylated at two cystein residues, Cys^26^ and Cys^29^, near the transmembrane domain in cytoplasmic region, and palmitoylated LAT is targeted constitutively to lipid rafts. Inhibition of palmitoylation on these cystein residues abrogates lipid raft targeting of LAT (12). However, it seems that LAT localization to lipid rafts is not essential during normal T cell activation and development. The reconstitution of LAT-deficient cell line with the LAX-LAT chimeric protein consisting of the cytoplasmic region of LAT fused with the transmembrane region of non-raft TRAP, LAX restored MAPK activation, calcium flux, and NFAT activation in LAT-deficient cells (21). Furthermore, the defects in thymocyte development and peripheral T cell responses in LAT-deficient mice are rescued by the reconstitution with the chimeric LAX-LAT protein (21). Therefore, it seems that the localization to lipid rafts may not directly affect the function of LAT in normal T cell activation and development. On the other hand, the raft localization of LAT may differentially affect its function in anergic T cells. It has been recently shown that palmitoylation of LAT is defective in anergic T cells (22). The early TCR signaling events such as CD3ξ-chain phosphorylation or Zap-70 phosphorylation are intact in these cells, however, tyrosine phosphorylation of LAT and calcium mobilization by PLCγ1 activation are significantly diminished. Interestingly, the palmitoylation defect in anergic T cells is likely specific to LAT, yet the mechanism is still unclear.

## LCK-INTERACTING TRANSMEMBRANE ADAPTOR PROTEIN (LIME)

Another raft-associated TRAP, LIME was identified as a positive regulator of immunoreceptor signaling (3,4). Originally, LIME was identified as a binding partner of Lck by yeast two hybrid screening (3). In Jurkat T cells, unlike LAT or NTAL, which are phosphorylated by Syk-family PTKs, LIME interact with and is phosphorylated by Lck upon TCR stimulation. Subsequently, LIME recruits the cytoplasmic proteins such as Lck, Fyn, Vav, p85 PI3K, Grb2, Gads, Shp-2, and Csk (3,4) to membrane proximal sites. In addition, LIME was shown to be phosphorylated by cross-linking of CD4 or CD8 coreceptors (4). LIME also possesses a dicystein palmitoylation motif and localize in the lipid rafts.

The binding partners of LIME are, in part, common with those of LAT, which include Gads, p85 PI3K, and Grb2 (3,13,14). When ectopically overexpressed in Jurkat T cells, LIME promotes TCR-mediated signaling pathways to drive calcium mobilization, MAPK activation, and IL-2 production (3). In contrast to the cell line studies, however, LIME-deficient mice show no significant alteration in the development of thymocytes, peripheral T cells, as well as other immune cells such as B cells, mast cells, and macrophages (Unpublished data, ref (23)). In addition, TCR-mediated responses such as T cell proliferation and cytokine production are normal in LIME-deficient mice, suggesting that LIME is dispensible in the development and activation of naïve T cells. The reason for the discrepancy between the results from cell line and knock-out mice studies is not clear at this stage. Unlike LAT, LIME expression is barely detectable in thymocytes and resting T cells but largely upregulated upon T cell activation (3). Thus, it is likely that LAT acts as a main signal transducer of pre-TCR and TCR signaling for the development of thymocytes and the activation of naïve peripheral T cells, whereas LIME may function in the later stage of T cell activation. It will be interesting to test whether the function of LIME is redundant to that of LAT in activated/effector T cells.

LIME is also expressed in B cells. In B cell lines, after its phosphorylation by Lyn, LIME augments BCR-mediated signaling pathways leading to activation of NFAT and NF-κB pathways (24). However, the experiments using LIME-deficient mice show that LIME is dispensable in the development and the function of B cells (23). Furthermore, the absence of LIME has no further effect on the autoimmune syndrome of NTAL-deficient mice (23). Interestingly, histological analysis reveals that LIME is expressed in plasma cells, as well as myeloma/plasmacytoma (25). Further studies are required to elucidate the functions of LIME especially in the effector T cells or plasma B cells.

## CONCLUSION

So far, both LAT and LIME were found to positively regulate TCR-mediated signaling events. However, unlike LAT-deficient mice, LIME-deficient mice are normal in the thymocyte development and peripheral T cell activation. Although the binding partners are partially overlapping, LAT and LIME also have several distinct characteristics. First, LAT is phosphorylated by Syk-family kinases, while LIME is phosphorylated by Lck. Second, the expression patterns of LAT and LIME are different. LAT is expressed starting from double negative stage of thymocytes and, in peripheral T cells, the level is constant regardless of the activation status. On the other hand, LIME expression is barely detectable in thymocytes and resting peripheral T cells and upregulated upon T cell activation and differentiation to effector T cells. In addition, LIME but not LAT is also expressed in B cells. Therefore, it is expected that the biological function of LAT and LIME are different. As LIME is induciblely expressed upon TCR engagement, it will be interesting to study the physiological significance of LIME in effector/memory T cells.

## Figures and Tables

**Table 1:**
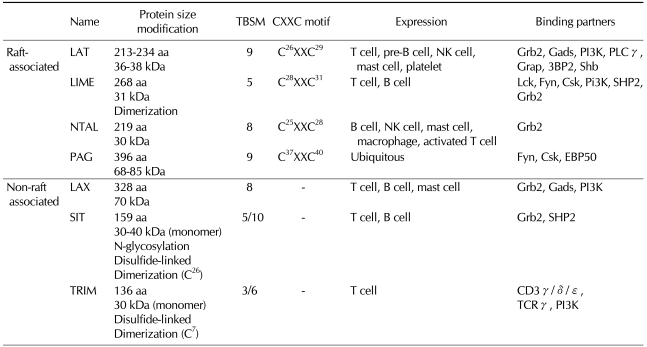
Characteristics of TRAPs
